# Cadmium Accumulation and Regulation in the Freshwater Mussel *Anodonta woodiana*

**DOI:** 10.3390/toxics13080646

**Published:** 2025-07-30

**Authors:** Xiubao Chen, Chao Song, Jiazhen Jiang, Tao Jiang, Junren Xue, Ibrahim Bah, Mengying Gu, Meiyi Wang, Shunlong Meng

**Affiliations:** 1 Freshwater Fisheries Research Center, Chinese Academy of Fishery Sciences, Wuxi 214081, China; 2 Wuxi Fisheries College, Nanjing Agricultural University, Wuxi 214081, China; 3 College of Science, China Agricultural University, Beijing 100193, China; 4 Ministry of Fisheries and Marine Resources, Freetown 190, Sierra Leone; 5 College of Marine Science and Technology and Environment, Dalian Ocean University, Dalian 116023, China

**Keywords:** cadmium, stable isotope dual-tracer technique, *Anodonta woodiana*, bioaccumulation, transcriptome, molecular regulatory

## Abstract

Cadmium (Cd) pollution poses a serious threat to freshwater ecosystems. The freshwater mussel *Anodonta woodiana* is increasingly used as a bioindicator for monitoring Cd pollution in aquatic environments. However, the primary routes of Cd accumulation in *A. woodiana* remain unclear, and the molecular regulatory mechanisms underlying Cd accumulation are poorly understood. To address these gaps, this study employed a novel stable isotope dual-tracer technique to trace Cd from water (waterborne ^112^Cd) and the green alga *Chlorella vulgaris* (dietary ^113^Cd) during the simultaneous exposure experiment. Comparative transcriptomic analysis was then conducted to characterize molecular responses in *A. woodiana* following Cd exposure. The results showed that although newly accumulated ^112^Cd and ^113^Cd increased with exposure concentration and duration, the relative importance of ^112^Cd (91.6 ± 2.8%) was significantly higher than that of ^113^Cd (8.4 ± 2.8%) (*p* < 0.05). Cd exposure induced differentially expressed genes primarily enriched in the metabolic processes, cellular processes, and/or the ubiquitin-mediated proteolysis pathway. Within the ubiquitin-mediated proteolysis pathway, TRIP12 (E3 ubiquitin-protein ligase TRIP12) and Cul5 (cullin-5) were significantly upregulated. The findings will provide critical insights for interpreting Cd biomonitoring data in freshwater environments using mussels as bioindicators.

## 1. Introduction

Cadmium (Cd) pollution turns out to be a global environmental problem [1,2]. It was ranked first among the 12 priority hazardous substances globally by the United Nations Environment Programme [3]. In freshwater environments, Cd pollution, primarily originating from mining, industrial activities, wastewater discharge, sedimentation, and agricultural practices, poses particularly severe risks [1,4]. For instance, China’s Xiangjiang River experienced a severe Cd pollution incident in 2006, causing Cd concentrations in the nearby water to exceed 25 times that of the national standard [5], and Cd was ranked 10th out of 71 common chemicals in terms of hazard risk in British rivers [1]. Furthermore, Cd in freshwater environments can threaten human health through biomagnification in the food chain [2], potentially causing bone disorders, kidney disease, and cancer [6]. Therefore, monitoring of Cd pollution dynamics in freshwater environments is of critical importance.

Biomonitoring serves as a crucial approach for assessing Cd pollution dynamics in freshwater environments [7,8]. Compared with conventional physicochemical approaches, biomonitoring provides distinct advantages: (1) enabling continuous Cd tracking; (2) reflecting Cd bioavailability in aquatic environments; (3) serving as an early warning indicator for Cd contamination due to its high bioaccumulation potential; and (4) offering cost-effectiveness for long-term monitoring programs [7,8]. The freshwater mussel *Anodonta woodiana*, originating from China’s Yangtze River basin, has now become widely distributed across Asia, Europe, North America, and Africa [9]. As a unique indicator species in the “Freshwater Mussel Watch” project [10,11], *A. woodiana* demonstrates hyperaccumulation characteristics for Cd [12]. Consequently, an increasing number of nations—including Poland, China, Serbia, and Bulgaria—have employed *A. woodiana* for biomonitoring Cd pollution in various freshwater environments such as rivers, lakes, and reservoirs [10,13,14,15]. However, the primary source of Cd accumulation in *A. woodiana* (whether waterborne or dietary) and its regulatory mechanisms remain poorly understood.

Cd has multiple stable isotopes (e.g., ^111^Cd, ^112^Cd, ^113^Cd, and ^114^Cd), all of which are suitable for tracer applications [16,17]. The emerging Cd stable isotope dual-tracer technique [16,17] enables the differentiation of the relative contribution rates of waterborne and dietary Cd accumulation in aquatic animals under co-exposure conditions (closer to natural aquatic environments). For example, Guo et al. [16] successfully employed this Cd isotope dual-tracer technique to characterize the gastrointestinal absorption patterns of waterborne versus dietary Cd in the marine yellowstripe goby (*Mugilogobius chulae*). Additionally, comparative transcriptome analysis can reveal the molecular regulatory mechanisms underlying Cd accumulation in aquatic organisms [18,19]. Zhao et al. [18] employed comparative transcriptomics to reveal that the hyperaccumulation capacity of Cd in the scallop *Chlamys farreri* is achieved through regulation of antioxidant defense, detoxification, and transport processes. Therefore, the stable isotope dual-tracer technique combined with comparative transcriptomic analysis shows significant potential for investigating both the sources and regulatory mechanisms of Cd accumulation in *A. woodiana*.

This study will first conduct a simultaneous exposure of *A. woodiana* to waterborne ^112^Cd and dietary ^113^Cd, followed by comparative transcriptome analysis to reveal (1) the accumulation dynamics of waterborne and dietary Cd in the mussel, and (2) the molecular regulatory mechanisms governing Cd accumulation. The findings will provide critical insights for interpreting Cd biomonitoring results in freshwater environments using mussels as bioindicators.

## 2. Materials and Methods

### 2.1. Experimental Animals and Metals

*Anodonta woodiana* (6-month-old; shell length: 5.7 ± 0.3 cm) were collected from the Freshwater Fisheries Research Center, Chinese Academy of Fishery Sciences (Wuxi, China). After removing epibionts from the shell surface, the mussels were acclimatized in aerated ASTM (American Society for Testing Materials) reconstituted soft water [20] at 20 °C under a 16:8 h light–dark cycle for 2 weeks. They were fed daily with 4 × 10^5^ cells/mL of the green alga *Chlorella vulgaris* for 3 h, followed by a complete water change.

The stable isotopes ^112^Cd (98.70% purity) and ^113^Cd (93.35% purity) were purchased from ISOFLEX USA (San Francisco, CA, USA) for labeling waterborne ^112^Cd and dietary ^113^Cd, respectively. The other Cd source was from CdCl_2_ (analytical grade; Sinopharm Chemical Reagent Co., Ltd., Shanghai, China) containing Cd with natural isotopic ratios.

### 2.2. Stable Isotope Cd Spiking in Water and Diet

ASTM reconstituted soft water was used as the experimental water [11,20]. In the control group, neither ^112^Cd nor ^113^Cd was detected in the water. In the ^112^Cd-spiked water, the nominal concentrations of ^112^Cd were 2.5, 5.0, and 10.0 μg L^−1^, while the actual measured concentrations were 2.5 ± 0.03, 5.3 ± 0.2, and 10.8 ± 0.5 μg L^−1^, which are environmentally relevant. In the ^112^Cd-spiked water, ^112^Cd accounted for >99% of the total Cd (^112^Cd + ^113^Cd), whereas ^113^Cd constituted <1% and was thus negligible. Therefore, ^112^Cd could well trace the waterborne Cd accumulation in mussels.

*Chlorella vulgaris* was suitable as a food source for *A. woodiana* [11]. In the control group, the ^112^Cd and ^113^Cd concentrations in *C. vulgaris* were 1.5 ± 0.01 and 0.8 ± 0.01 μg g^−1^ dw, respectively, which are comparable to Cd levels in algae from natural aquatic environments [21]. To obtain ^113^Cd-labeled *C. vulgaris*, the algae were exposed to ^113^Cd solutions with nominal concentrations of 2.5, 5.0, and 10.0 μg L^−1^ for 7 d [22]. The ^113^Cd-spiked algae were then collected via centrifugation and washed three times with Milli-Q water (Millipore Corp., Burlington, MA, USA, with a resistivity of 18.2 MΩ·cm). After labeling, the ^112^Cd concentrations in the algae showed no significant change (1.4–1.6 μg g^−1^ dw), whereas the ^113^Cd concentrations increased to 1.9 ± 0.01, 3.7 ± 0.1, and 6.2 ± 0.2 μg g^−1^ dw, representing 2.3- to 7.4-fold increases compared to the control group. These results demonstrate that ^113^Cd could effectively trace the dietary Cd accumulation in mussels. The collected ^113^Cd-spiked algae were stored at −80 °C until they were fed to mussels.

### 2.3. Simultaneous Exposure to Waterborne ^112^Cd and Dietary ^113^Cd

A total of 180 mussels were simultaneously exposed to waterborne ^112^Cd and dietary ^113^Cd and randomly divided into four groups: the control group (T0, 0 μg L^−1^ waterborne ^112^Cd + normal algae), low-Cd treatment group (T1, 2.5 μg L^−1^ waterborne ^112^Cd + 1.9 μg g^−1^ dw dietary ^113^Cd), medium-Cd treatment group (T2, 5.3 μg L^−1^ waterborne ^112^Cd + 3.7 μg g^−1^ dw dietary ^113^Cd), and high-Cd treatment group (T3, 10.8 μg L^−1^ waterborne ^112^Cd + 6.2 μg g^−1^ dw dietary ^113^Cd). Based on previous rearing density [11], the mussels were randomly distributed into 12 glass tanks (3 replicates per group), each containing 3 L of test solution and 15 mussels. They were fed daily with 4 × 10^5^ cells mL^−1^ [11,23] of *C. vulgaris* for a 3 h feeding period [11,24]. This duration was selected because (1) according to the filtration rate of *A. woodiana* [25], 3 h allowed the mussels to filter the tank water 3–4 times, ensuring sufficient algal uptake, and (2) it minimized the potential release of Cd from algae into the water or absorption of Cd from the water by algae [24]. After feeding, complete water replacement was performed. The 30 d treatment was conducted under continuous aeration, with water temperature maintained at 20 °C and a 16:8 h light–dark cycle. On days 10, 20, and 30, 6 mussels per group (2 per tank) were sampled. The remaining specimens were reserved for further studies (unpublished).

### 2.4. Transcriptome Analysis

The mussels were exposed to waterborne Cd with nominal concentrations of 2.5, 5.0, and 50 μg L^−1^, while the actual measured concentrations were 2.6 ± 0.1, 5.5 ± 0.2, and 56 ± 1.6 μg L^−1^, respectively. The first two Cd concentrations were environmentally relevant, whereas the highest concentration was set to comprehensively investigate the molecular regulatory mechanisms of Cd accumulation in the mussels. Additionally, ASTM reconstituted soft water containing 0 μg L^−1^ Cd served as the control group. A total of 120 mussels were randomly and equally divided into the four groups mentioned above, with three replicates per group. The stocking density and exposure conditions followed Section 2.3. After 10 d of exposure, the gills—the target organ for Cd accumulation [11]—were collected from different groups (*n* = 3) for transcriptomic analysis, as previously described [26]. Briefly, total RNA was extracted using RNAiso Plus and sequenced on the Illumina NovaSeq 6000 platform. The raw sequence data were deposited in the NCBI database under the project number PRJNA760280. After trimming and filtering, clean reads were de novo assembled using Trinity software (v2.4.0) [27]. Subsequently, gene annotation was performed using the Nr, COG, GO, KEGG, KOG, Pfam, and Swiss-Prot databases. Differentially expressed genes (DEGs) were identified using DESeq2 software (v 1.22.2) with the thresholds of |log_2_(fold change)| > 1 and false discovery rate < 0.05 [28]. The DEGs were further subjected to the GO enrichment and KEGG pathway analyses.

To validate the reliability of the transcriptomic data, 30 mussels were equally divided into a control group (0 μg Cd L^−1^) and an exposure group with a nominal Cd concentration of 2.0 mg L^−1^ (actual measured concentration: 2.2 ± 0.1 mg L^−1^; approximately half the 96 h EC_50_ value [29]), with three replicates per group. Each glass tank contained 2 L of test solution and five mussels. With the exception of food deprivation, all other exposure conditions were identical to those described in Section 2.3. After 96 h of exposure, the gills from different treatment groups (*n* = 3) were sampled for real-time PCR (RT-qPCR) analysis of four representative genes. Gene-specific primers are listed in Table 1. The RT-qPCR was performed as previously described [26], with β-actin serving as the internal control [26]. Relative gene expression levels were calculated using the 2^−ΔΔCT^ method [30].

### 2.5. Heavy Metal Analysis

Water samples (30 mL) were acidified with 1.5 mL of HNO_3_ (Merck, Darmstadt, Germany, 65%). For algal and soft tissue samples of *A. woodiana*, specimens were dried at 80 °C for 24 h, ground into powder, and approximately 0.1 g was digested with 5 mL HNO_3_ using an ETHOS A T260 microwave digestion system (Milestone Inc., Milan, Italy). The digested solutions were then diluted to 100 mL with Milli-Q water. The concentrations of ^112^Cd, ^113^Cd, and Cd in water, algal, and mussel samples were determined by an Agilent 7500ce inductively coupled plasma mass spectrometry (ICP-MS; Agilent Technologies, Santa Clara, CA, USA). Quality assurance and quality control of the ICP-MS were checked by spike recoveries of ^112^Cd, ^113^Cd (ISOFLEX USA, San Francisco, CA, USA), and certified reference materials (Agilent Technologies, Santa Clara, CA, USA), with all recovery rates showing deviations of less than 10% from the certified values.

### 2.6. Statistical Analysis

The accumulation of waterborne ^112^Cd in mussels was expressed as the ^112^Cd concentration in mussels measured by ICP-MS, subtracting the ^112^Cd absorbed from ^113^Cd-spiked algae. The accumulation of dietary ^113^Cd in mussels was calculated by multiplying the ^113^Cd concentration in mussels measured via ICP-MS by 8 (3 h × 8 = 24 h), as they continuously filter water and feed over a 24 h period. The newly accumulated waterborne ^112^Cd and dietary ^113^Cd in *A. woodiana* were calculated by subtracting control group concentrations from exposure group concentrations. The influx rates (*J*_in_, μg g^−1^ d^−1^) of waterborne ^112^Cd and dietary ^113^Cd were calculated by linear regressions between the new accumulation of ^112^Cd or ^113^Cd and exposure time [16]. All data were presented as mean ± SD. Statistical analyses were performed using SPSS Statistics 29.0 (IBM Corporation, Armonk, NY, USA), including one-way ANOVA and *t*-tests, with *p* < 0.05 considered significant difference.

## 3. Results

### 3.1. Survival and Growth of Mussels

During the 30 d simultaneous exposure to waterborne ^112^Cd and dietary ^113^Cd, the survival rate of mussels in both the control group and all treatment groups was 100%. The shell length ranged from 6.81 ± 0.23 cm to 7.43 ± 0.21 cm (Table 2), while the dry weight of soft tissues varied from 0.73 ± 0.07 g to 1.29 ± 0.20 g (Table 2). No significant differences were observed in shell length or dry weight of soft tissues among different groups (*p* > 0.05).

### 3.2. Cadmium Accumulation in Mussels from Waterborne and Dietary Exposure

**Figure 1:**
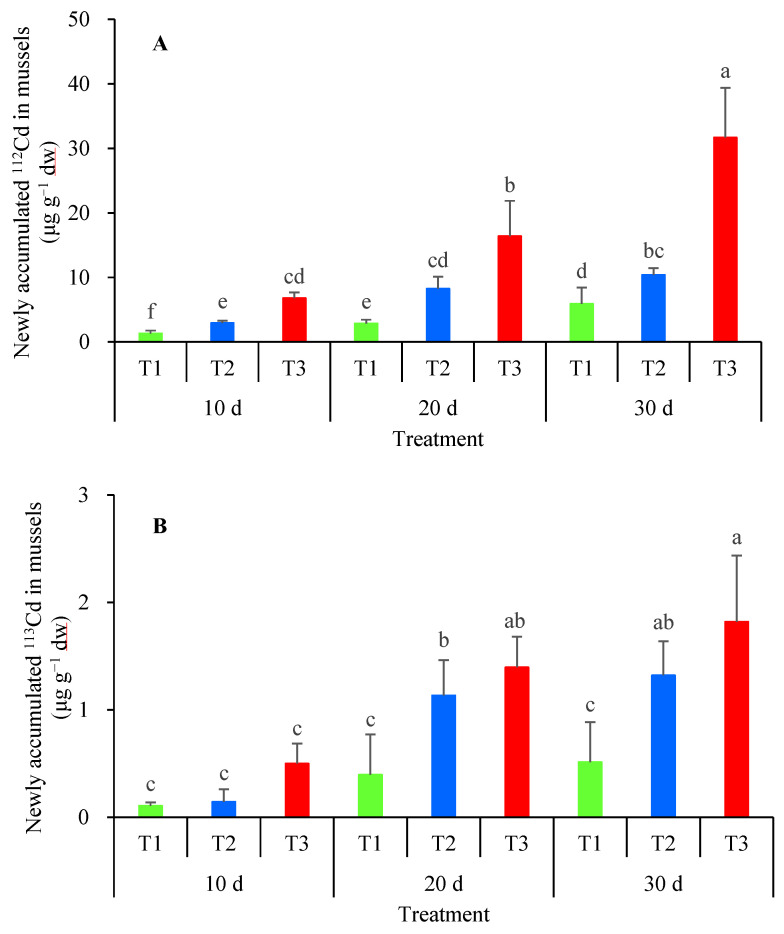
The newly accumulated ^112^Cd (**A**) and ^113^Cd (**B**) in *Anodonta woodiana* simultaneously exposed to waterborne ^112^Cd and dietary ^113^Cd over 30 d (*n* = 3). T1 refers to 2.5 μg L^−1^ waterborne ^112^Cd and 1.9 μg g^−1^ dw dietary ^113^Cd simultaneous exposure (green); T2 refers to 5.3 μg L^−1^ waterborne ^112^Cd and 3.7 μg g^−1^ dw dietary ^113^Cd simultaneous exposure (blue); and T3 refers to 10.8 μg L^−1^ waterborne ^112^Cd and 6.2 μg g^−1^ dw dietary ^113^Cd simultaneous exposure (red). Values with different letters indicate a significant difference (*p* < 0.05).

In the control group, the concentrations of ^112^Cd and ^113^Cd in mussels were 1.52 ± 0.02 μg g^−1^ dw (range: 1.45–1.59 μg g^−1^ dw) and 0.87 ± 0.04 μg g^−1^ dw (range: 0.83–0.91 μg g^−1^ dw), respectively. In the treated group, the newly accumulated ^112^Cd in mussels increased with rising waterborne ^112^Cd concentration and prolonged exposure time (Figure 1A). The *J*_in_ of waterborne ^112^Cd was 0.62 ± 0.55 μg g^−1^ d^−1^ (range: 0.23–1.25 μg g^−1^ d^−1^). Similarly, the newly accumulated ^113^Cd in mussels increased with higher dietary ^113^Cd concentration and extended exposure duration (Figure 1B). The *J*_in_ of dietary ^113^Cd was 0.05 ± 0.02 μg g^−1^ d^−1^ (range: 0.02–0.07 μg g^−1^ d^−1^). In the treated group, the relative importance of ^112^Cd was 91.6 ± 2.8% (range: 87.9–95.3%), whereas that of ^113^Cd was 8.4 ± 2.8% (range: 4.7–12.1%). The former was significantly higher than the latter (*p* < 0.05).

### 3.3. Transcriptomic Response of Mussels to Cd Accumulation

**Figure 2:**
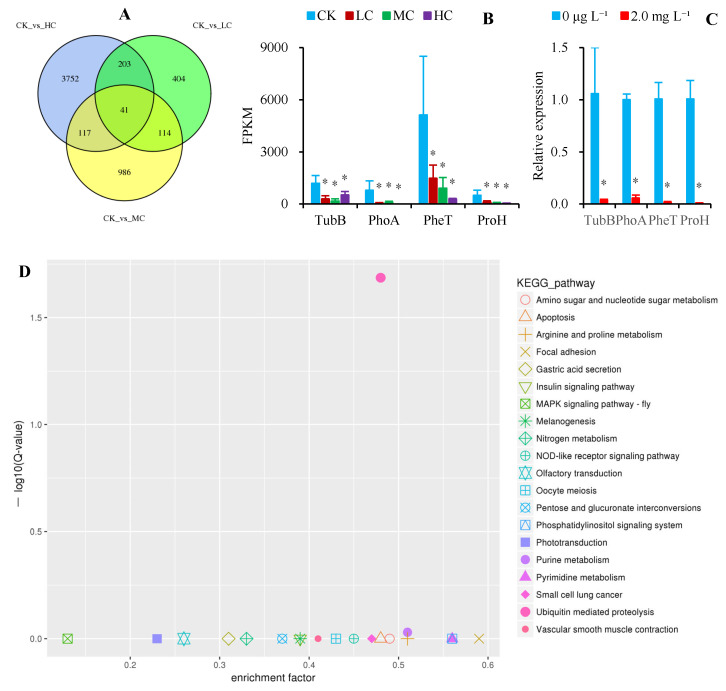
(**A**) Differentially expressed genes (DEGs) induced by waterborne Cd exposure at 2.5 μg L^−1^ (low concentration, LC), 5.0 μg L^−1^ (medium concentration, MC), and 50 μg L^−1^ (high concentration, HC) in *Anodonta woodiana* for 10 d compared to the control group (0 μg L^−1^, CK); (**B**) RNA-Seq and (**C**) RT-qPCR validation of relative expression levels for the genes TubB (Tubulin beta chain), PhoA (Phospholipase A1 magnifin), PheT (Phenylalanine-tRNA ligase), and ProH (Protein henna); and (**D**) KEGG pathway analysis of DEGs. Superscript asterisks indicate significant differences between the Cd-treated and control groups (*p* < 0.05).

Exposure of mussels to waterborne Cd at low (LC; 2.5 μg L^−1^), medium (MC; 5.0 μg L^−1^), and high concentrations (HC; 50 μg L^−1^) for 10 d resulted in 762, 1258, and 4113 DEGs, respectively, compared to the control group (CK; 0 μg L^−1^). Among these, 41 common DEGs were identified (Figure 2A). Four genes were selected from these forty-one common DEGs for qRT-PCR validation, which confirmed consistent expression trends with the transcriptomic data (Figure 2B,C). The GO enrichment analysis revealed that the DEGs were predominantly enriched in biological processes such as metabolic processes (GO:0044710) and cellular processes (GO:0044763). The KEGG pathway analysis indicated no significant enrichment of DEGs in the LC and MC groups, whereas the HC group exhibited significant enrichment in the ubiquitin-mediated proteolysis pathway (ko04120; Figure 2D). Within this pathway, TRIP12 (E3 ubiquitin-protein ligase TRIP12) and Cul5 (cullin-5) were significantly upregulated, while UBE2D (ubiquitin-conjugating enzyme E2-17 kDa) and EloC (Elongin C) were significantly downregulated.

## 4. Discussion

### 4.1. Effect of Cd Exposure on A. woodiana

The freshwater mussel *A. woodiana* exhibits high tolerance to Cd. In natural aquatic environments, the accumulated Cd concentration in its soft tissues can reach up to 126.2 ± 56.9 μg g^−1^ dw [12], which may be attributed to its detoxification mechanisms involving the production of antioxidant enzymes [31] and/or metallothioneins for Cd sequestration [32]. Due to this tolerance, no mortality was observed in *A. woodiana* after 30 d of simultaneous exposure to environmentally relevant concentrations of waterborne ^112^Cd and dietary ^113^Cd. Nevertheless, no significant growth (including shell length and soft tissue weight) was detected during the exposure period. This also eliminated the potential influence of the growth dilution effect [33] on Cd accumulation.

### 4.2. Accumulation Characteristics of Waterborne and Dietary Cd in A. woodiana

The uptake and accumulation of both waterborne and dietary Cd in mussels may be influenced by their background Cd levels [34]. For instance, the green mussel *Perna viridis* exhibits a significant positive linear correlation between its dietary Cd assimilation efficiency and background Cd concentration [34]. However, excessively high background Cd concentrations (22.5 ± 2.5 μg g^−1^ dw) can significantly inhibit waterborne Cd uptake [34]. Based on the natural abundance of ^112^Cd (24.13%) and ^113^Cd (12.22%) in Cd [35], the background Cd concentration in *A. woodiana* used in this study was much lower than the maximum permissible Cd level for bivalves (2.0 μg g^−1^ wet weight, equivalent to approximately 20 μg g^−1^ dw) specified in China’s National Food Safety Standard—Limits of Contaminants in Foods (GB 2762-2022) [36]. Therefore, the influence of background Cd levels on the uptake and accumulation of waterborne ^112^Cd and dietary ^113^Cd could be negligible in this study.

In Cd-treated groups of *A. woodiana*, the accumulated concentrations of ^112^Cd and ^113^Cd increased with elevated exposure concentrations and/or prolonged exposure duration, demonstrating active Cd uptake from both waterborne and dietary pathways. Notably, the influx rate of waterborne ^112^Cd was significantly higher than that of dietary ^113^Cd, with the former being 12.4-fold greater than the latter on average. Correspondingly, the mean relative importance of ^112^Cd accumulation was 10.9 times higher than that of ^113^Cd, indicating that the majority of accumulated Cd originated from water. Previous studies have consistently demonstrated that *A. woodiana* exhibits high efficiency in absorbing waterborne Cd across both environmentally relevant [11] and elevated concentration [37] ranges. Specifically, the average waterborne Cd uptake rate in *A. woodiana* exceeded that of the goby *Mugilogobius chulae* (mean: 0.12 μg g^−^1 d^−1^, range: 0.031–0.24 μg g^−1^ d^−1^) by more than 5-fold [38]. In the freshwater mussel *Pyganodon grandis*, gills served as the primary site for waterborne Cd uptake (waterborne-to-dietary Cd ratio: 99:1), while the digestive gland was the main route for dietary Cd assimilation, waterborne Cd still dominated (ratio: 80:20) [23]. These findings collectively suggest that when freshwater bivalves like *A. woodiana* are employed as bioindicators for monitoring Cd contamination in aquatic environments, they primarily reflect waterborne rather than dietary Cd concentrations.

### 4.3. Molecular Mechanisms of Cd Accumulation Regulation in A. woodiana

This study demonstrates that waterborne Cd is the primary route for Cd accumulation in *A. woodiana* from the aquatic environment. Therefore, we further investigated the molecular mechanisms regulating Cd accumulation in *A. woodiana* under waterborne Cd exposure.

Waterborne Cd accumulation induces oxidative stress in *A. woodiana* [31,39]. This study revealed that the number of DEGs in *A. woodiana* increased with rising waterborne Cd concentrations. These DEGs were primarily associated with metabolic and cellular processes. Previous studies have also found that waterborne Cd exposure disrupts nucleotide, amino acid, and energy metabolism in the deep-sea mussel *Bathymodiolus platifrons* [40], and triggers apoptosis in *A. woodiana* [39]. In response, *A. woodiana* upregulates antioxidant enzymes (e.g., superoxide dismutase, glutathione peroxidase, and catalase) to counteract Cd-induced oxidative stress [31]. Notably, this study newly identified that *A. woodiana* regulates Cd accumulation via the ubiquitin-mediated proteolysis pathway, which is linked to the antioxidant system [41]. The ubiquitin-mediated proteolysis pathway has also been reported as a key regulatory pathway for waterborne Cd detoxification in the clam *Ruditapes philippinarum* [19]. Ubiquitin-dependent proteolysis resists Cd by degrading abnormal proteins [42]. Specifically, we found that TRIP12, an ubiquitination-mediated degradation of targeting protein substrates [43], was significantly upregulated in the ubiquitin-mediated proteolysis pathway. By facilitating the removal of damaged proteins, TRIP12 participates in vital biological processes, including cell cycle progression, DNA damage repair, chromatin remodeling, and cell differentiation [43]. Therefore, we propose that upregulation of TRIP12 in the ubiquitin-mediated proteolysis pathway serves as a crucial mechanism for *A. woodiana* to regulate Cd accumulation.

## 5. Conclusions

The freshwater mussel *A. woodiana* is being increasingly utilized for biomonitoring of Cd pollution in aquatic environments. This study reveals that the predominant source of Cd accumulation in *A. woodiana* originates from waterborne rather than dietary influx, with waterborne Cd exhibiting approximately 10-fold greater relative importance compared to dietary Cd. The mussel regulates Cd accumulation through modulation of metabolic processes, cellular processes, and/or the ubiquitin-mediated proteolysis pathway. Significantly, upregulation of TRIP12 was identified as a key mechanism in this regulatory process. These findings provide crucial insights for interpreting biomonitoring results of aquatic Cd pollution using freshwater mussels and will aid global Cd pollution assessments. Future studies should further investigate the Cd accumulation kinetics in *A. woodiana* based on the current findings. Moreover, since pollutants in natural waters typically consist of multi-component mixtures rather than Cd alone, subsequent research should also explore the interaction mechanisms between Cd accumulation and other coexisting elements (including both essential and non-essential elements) in *A. woodiana*.

## Figures and Tables

**Table 1:** Primers used in the real-time PCR analysis.

Gene	Description	Primer Sequence
TubB	Tubulin beta chain	F-AGGCAAATATGTACCCAG
		R-TTTCCAGCTCCACTCTGT
PhoA	Phospholipase A1 magnifin	F-AGCACCCTTATTCCAGTC
		R-CAGATAGAAACGTCGTATTG
PheT	Phenylalanine-tRNA ligase	F-TGCAATATGATGAAAGATAC
		R-ACTTGCTGTACGAAGTCA
ProH	Protein henna	F-TTTGACACGGTGAAGGAC
		R-ACGGGATTCAATGTGGAG
β-actin	Beta-actin	F-ACGGATAACACAAGGAAAGGAAAC
		R-ATGGATGGAAACACGGCTCT

**Table 2:** Growth characteristics of *Anodonta woodiana* (mean ± SD; *n* = 3).

Time (d)	Treatment	Shell Length (cm)	Dry Weight of Soft Tissue (g)
10	T0	7.43 ± 0.21	1.29 ± 0.20
	T1	7.08 ± 0.07	1.18 ± 0.36
	T2	6.94 ± 0.29	1.23 ± 0.32
	T3	6.90 ± 0.10	1.06 ± 0.17
20	T0	7.02 ± 0.16	1.22 ± 0.29
	T1	7.08 ± 0.20	1.14 ± 0.31
	T2	6.93 ± 0.52	0.92 ± 0.04
	T3	6.81 ± 0.23	0.86 ± 0.10
30	T0	7.18 ± 0.45	1.00 ± 0.16
	T1	6.98 ± 0.54	0.73 ± 0.07
	T2	6.91 ± 0.08	0.82 ± 0.07
	T3	7.02 ± 0.27	1.00 ± 0.23

Note: T0 refers to 0 μg L^−1^ waterborne ^112^Cd and 0.8 μg g^−1^ dw dietary ^113^Cd simultaneous exposure; T1 refers to 2.5 μg L^−1^ waterborne ^112^Cd and 1.9 μg g^−1^ dw dietary ^113^Cd simultaneous exposure; T2 refers to 5.3 μg L^−1^ waterborne ^112^Cd and 3.7 μg g^−1^ dw dietary ^113^Cd simultaneous exposure; and T3 refers to 10.8 μg L^−1^ waterborne ^112^Cd and 6.2 μg g^−1^ dw dietary ^113^Cd simultaneous exposure.

## Data Availability

The original contributions presented in this study are included in the article. Further inquiries can be directed to the corresponding author.
